# Cyclogram analysis of canine gait: quantifying inter-joint coordination and clinical utility in rehabilitation

**DOI:** 10.3389/fvets.2026.1783495

**Published:** 2026-04-10

**Authors:** Kazuyuki Yoshikawa, Taisuke Iwata, Shintaro Tomura, Atsushi Fujita, Kosuke Haii, Kazuya Edamura, Akio Shimada, Tsuyoshi Kadosawa

**Affiliations:** 1 Japan Small Animal Medical Center, Tokorozawa, Japan; 2 Graduate School of Veterinary Medicine, Rakuno Gakuen University, Ebetsu, Hokkaido, Japan; 3 Laboratory of Veterinary Surgery, Department of Veterinary Medicine, College of Bioresource Sciences, Nihon University, Fujisawa, Kanagawa, Japan; 4 Togasaki Animal Hospital, Misato, Japan; 5 Toyo Sogu, Machida, Japan

**Keywords:** canine, cyclogram, femoral head and neck ostectomy, gait analysis, hindlimb kinematics, inter-joint coordination, quantitative outcome measures, veterinary physical rehabilitation

## Abstract

**Introduction:**

Cyclograms, or joint angle–angle plots, provide an intuitive representation of inter-joint coordination, yet they remain underused in canine rehabilitation. This study established sagittal-plane hindlimb cyclograms in dogs and evaluated the absolute enclosed cyclogram area (A_abs, deg²) as a reproducible and clinically interpretable index of coordination.

**Methods:**

Eight clinically healthy Beagles were recorded during treadmill walking, and hip–stifle and tarsus–stifle cyclograms were generated from 10 consecutive gait cycles per dog. A_abs was calculated using a polygonization-based approach applied to the cyclogram trajectory. Within-session repeatability was assessed using ICC(1,1) and ICC(1,10), together with measurement-error indices (SEM and MDC95). Clinical feasibility was illustrated in a client-owned dog undergoing rehabilitation after unilateral femoral head and neck ostectomy (FHNO), assessed during overground walking on postoperative days (PODs) 14, 21, and 31, with 10 gait cycles analyzed per session.

**Results:**

In healthy Beagles, the hip–stifle cyclogram formed a broad counterclockwise loop, whereas the tarsus–stifle cyclogram exhibited a characteristic figure-of-eight pattern. Mean A_abs was 813.18 ± 114.88 deg² for hip–stifle and 619.88 ± 129.00 deg² for tarsus–stifle. Stride-level repeatability was moderate [ICC(1,1) = 0.59 and 0.67], whereas the mean of 10 cycles showed excellent repeatability [ICC(1,10) = 0.93 and 0.95], with small MDC95 values for the 10-cycle mean (81.55 and 78.23 deg²). In the FHNO dog, cyclogram loops were initially tall and narrow on POD14, reflecting stifle-dominant motion with restricted hip and tarsal excursions, and became progressively wider and smoother by POD31. Consistently, mean A_abs increased from POD14 to POD31 (hip–stifle, 112.24 to 395.09 deg²; tarsus–stifle, 185.73 to 543.78 deg²), whereas stride-to-stride variability decreased (coefficient of variation, 66.76% to 30.61% and 77.32% to 42.18%).

**Discussion:**

These findings indicate that hindlimb cyclograms provide a compact visual summary of inter-joint coordination and that A_abs offers a reproducible quantitative descriptor in healthy dogs. In a single FHNO case, this metric also illustrated feasibility for tracking early postoperative gait changes in a clinical rehabilitation setting.

## 1 Introduction

Gait evaluation in dogs is routinely performed in veterinary practice; however, many assessments rely heavily on subjective judgments and observer experience, underscoring the need for more objective and reproducible evaluation methods. In recent decades, kinetic (force-plate) and kinematic gait analysis systems have been developed as objective tools that provide quantitative, repeatable measurements of normal and abnormal canine gait and have been widely adopted for orthopedics and rehabilitation of small animals (1–3). Traditional kinematic gait analyses in veterinary medicine have primarily focused on changes in joint angles over time in individual joints. However, gait is inherently a complex motion involving coordinated interactions between multiple joints; thus, assessment methods that explicitly account for inter-joint coordination are essential (4, 5).

In human medicine, cyclograms (planar angle-angle diagrams) are extensively utilized to evaluate inter-joint coordination during gait (6, 7). Cyclograms have proven particularly valuable for assessing pathological gait patterns associated with conditions such as stroke, osteoarthritis, and Parkinson’s disease (8–10). By simultaneously plotting the angular displacements of the two joints, cyclograms allow both visual and quantitative assessments of joint coordination during locomotion (7, 11). Beyond such qualitative assessments, recent studies have used hip–knee cyclograms to calculate simple geometric features, such as loop shape, orientation, enclosed area, and perimeter, and have shown that these parameters can classify disease groups and may help predict progression in conditions such as adolescent idiopathic scoliosis and knee osteoarthritis. These geometric variables also provide convenient summary indices of inter-joint coordination that are directly derived from the cyclogram geometry (12–14). Cyclograms can visually illustrate multi-joint interactions that may not be adequately captured by traditional angle-time analyses.

However, the application of cyclograms in veterinary gait analysis remains limited, particularly in canine studies. Notable exceptions include the pioneering canine implementation of the cyclographic technique (15) and a later investigation that employed joint angle–angle diagrams to assess locomotor stability at different speeds (16). In addition, angle–angle diagrams have been applied in other veterinary species; for example, high-speed videography was used to generate angle–time and angle–angle diagrams in sound Thoroughbreds, and the angle–angle diagrams were reported to be highly repeatable over multiple strides while also revealing varying degrees of right–left asymmetry (17). More recently, intralimb joint angle–angle cyclograms have been reported as coordination descriptors in veterinary animal models (e.g., sheep hindlimb cyclograms) (18). Sagittal-plane kinematic studies have described hip, stifle, and tarsal joint trajectories during walking and trotting in healthy and lame dogs (19–28), including recent three-dimensional fluoroscopic analyses that emphasize complex coupling among pelvic limb segments (26–28). However, these analyses generally treat each joint in isolation, focusing on angle–time waveforms or discrete kinematic variables rather than explicitly quantifying patterns of inter-joint coordination. Consequently, most veterinary gait analyses continue to rely primarily on angle–time kinematics or alternative non-graphical coordination metrics, and cyclogram-derived features remain largely unexplored in canine biomechanics. Bridging this methodological gap by adopting well-established cyclogram analysis frameworks from human medicine may enhance the objectivity and quantitative precision of inter-joint coordination assessments in veterinary biomechanics.

Femoral head and neck ostectomy (FHNO) is widely used as a salvage procedure to relieve intractable hip pain associated with severe hip joint diseases in dogs. However, long-term functional recovery remains heterogeneous, and several force plate studies have demonstrated subtle kinetic asymmetries in at least some dogs judged as clinically sound (29–33). In addition, perioperative factors, including postoperative physiotherapy, are associated with substantial variability in functional outcomes after FHNO (33–35). Therefore, the present study aimed to establish a foundational framework for cyclogram-based gait evaluation in dogs by addressing methodological validation in healthy dogs and illustrative clinical feasibility. Specifically, we (i) characterized the canonical planar cyclograms of healthy beagles walking on a treadmill by applying a canine gait-phase classification to delineate phase-specific coordination features that earlier approaches have not described in detail and quantified within-session repeatability and measurement error of the cyclogram area index (A_abs) using ICC and SEM/MDC95, and (ii) presented a single FHNO case as a preliminary illustrative example of within-dog longitudinal monitoring, investigating whether cyclogram geometry and A_abs can track postoperative changes during rehabilitation. We quantified cyclogram area using a cancellation-robust, polygonization-based definition (Route A). We aimed to validate repeatability of A_abs in healthy dogs and to present a single FHNO case as an illustrative example of within-dog longitudinal tracking. Because of marked between-sample heterogeneity, we avoided formal between-sample inference. Together, these results provide a methodological basis for cyclogram-derived indices and support feasibility for clinical rehabilitation use.

## 2 Materials and methods

### 2.1 Ethics approval and consent to participate

All procedures involving healthy beagles were conducted in accordance with institutional and national ethical guidelines and were approved by the Experimental Animal Research Committee of Rakuno Gakuen University (approval ID: VH17B7).

A longitudinal single-case evaluation following unilateral FHNO was undertaken as part of routine clinical care at the Japan Small Animal Medical Center. The owner of the animal provided written informed consent for the participation and use of anonymized clinical data for research and publication.

### 2.2 Study design

This study comprised two components: (i) a cross-sectional reference dataset from healthy beagle dogs, and (ii) a longitudinal single-case study following unilateral FHNO.

A healthy cohort was used to construct canonical hindlimb cyclograms and qualitatively describe typical inter-joint coordination patterns. In addition, to quantify within-session methodological repeatability in the healthy cohort, we computed stride-wise cyclogram area indices and quantified within-session repeatability using an intraclass correlation coefficient (ICC) framework and associated measurement-error indices (SEM and MDC95) (see Section 2.7.1).

The FHNO case was used to quantify longitudinal changes in the inter-joint coordination using geometric descriptors derived from cyclograms. As this study was exploratory in nature and was designed as a proof-of-concept study, we focused on interpreting cyclogram metrics within each component and avoided formal between-sample inferential comparisons between the healthy cohort and the FHNO case. Accordingly, we interpreted the FHNO results as within-dog longitudinal descriptors. To explore whether cyclogram-derived A_abs values in a single clinical case fall outside the distribution observed in the small healthy reference sample, we additionally performed a single-case vs. controls comparison using a modified *t*-test (Crawford–Howell) applied to dog-level healthy summary values; given the pronounced between-group methodological heterogeneity, these analyses were treated as exploratory only (see Section 2.7.3).

Accordingly, the primary contribution of this work is methodological validation and characterization in healthy dogs, while the FHNO case is presented as a preliminary illustrative example of longitudinal within-dog monitoring.

### 2.3 Animals, eligibility criteria, and rehabilitation protocol

#### 2.3.1 Healthy cohort

Eight clinically healthy beagles (three females and five males) owned by Rakuno Gakuen University were included. Mean ± standard error of the mean body mass was 12.3 ± 0.6 kg, and age ranged from 4 to 8 years (mean ± standard error of the mean, 5.6 ± 0.6 years). All dogs were confirmed to be healthy and free of orthopedic and neurological abnormalities based on standardized orthopedic and neurological examinations. The healthy sample size (*n* = 8) was chosen in line with previous canine treadmill kinematic studies, which typically characterized reference joint angle trajectories and coordination patterns using small cohorts of approximately 5–10 dogs (19–21).

#### 2.3.2 FHNO case

One client-owned toy poodle (intact male, 1 year of age, body mass 2.3 kg) that underwent unilateral right-sided FHNO for Legg–Calvé–Perthes disease was evaluated on postoperative days (POD) 14, 21, and 31 during a standardized postoperative rehabilitation program with a prescribed home-exercise and home-modality plan initiated at discharge.

The postoperative rehabilitation followed a structured protocol adapted from previously described canine FHNO rehabilitation protocols (34–36). During the first three POD, the dog underwent in-hospital rehabilitation twice daily (30-min sessions in the morning and afternoon), consisting of cryotherapy, therapeutic laser treatment (H1; Summus Medical Laser, Franklin, TN, USA), passive range of motion exercises, slow leash walking, and basic balance exercises. At discharge (POD4), the owner was instructed to perform home exercises (PROM/hip flexion–extension stretching, 10–15 repetitions BID–TID; balance exercises on a foam pad BID–TID) and slow, controlled leash walking (5–20 min) including mild inclines, while avoiding running/jumping and slippery floors. Home modalities included heat therapy prior to exercise (10–15 min BID–TID; avoided if signs of acute inflammation were present) and cryotherapy following exercise (15–20 min BID). Postoperative analgesia consisted of firocoxib (Previcox; 57 mg tablet, 1/4 tablet PO q24h) for 4 days only; therefore, no analgesics were continued during gait data acquisition at POD14/21/31. After hospital discharge, outpatient rehabilitation was provided approximately once weekly in 50-min sessions, with stepwise progression of these modalities. Exercise intensity and complexity were adjusted according to the dog’s clinical status, comfort, and limb use. As recovery progressed, weight-bearing and dynamic balance tasks progressively increased, and controlled treadmill walking, cavaletti rail exercises, and sit-to-stand exercises were incorporated into the program.

### 2.4 Experimental setup and data acquisition

#### 2.4.1 Healthy beagle cohort

Kinematic recordings of healthy beagle dogs walking were performed on a treadmill (AF1900; ALINCO, Osaka, Japan). The dogs were allowed to habituate to treadmill walking, and data were collected once each dog walked comfortably at a speed of 0.7 m/s. Ten valid gait cycles were recorded from each dog. Hindlimb motion was recorded using a three-dimensional motion analysis system (Kinema Tracer; Kissei Comtec Co., Ltd., Matsumoto, Japan) at 60 Hz. Kinematic recordings and subsequent analyses were performed for the left hindlimb only, and the analyzed side was consistent across all dogs. Bilateral recordings and right–left comparisons were not performed in this dataset.

#### 2.4.2 FHNO case

In the FHNO case, sagittal-plane video recordings were obtained during overground walking at a comfortable self-selected speed. The recordings were conducted in a dedicated rehabilitation room that provided a quiet environment, was free of other animals, and offered sufficient space for straight walking. After the dog became habituated to straight walking on the mat, 10 valid gait cycles were acquired at each postoperative time point. Hindlimb joint angles were analyzed using a two-dimensional motion analysis system (ICpro-2D; Hutech Co., Ltd., Tokyo, Japan) at 60 Hz. Joint angles and cyclograms were derived from the operated (right) hindlimb only; contralateral hindlimb kinematics were not recorded/digitized in this proof-of-concept case.

### 2.5 Marker placement, joint-angle definitions, gait phases, and preprocessing

#### 2.5.1 Marker placement and joint-angle model

**Figure 1:**
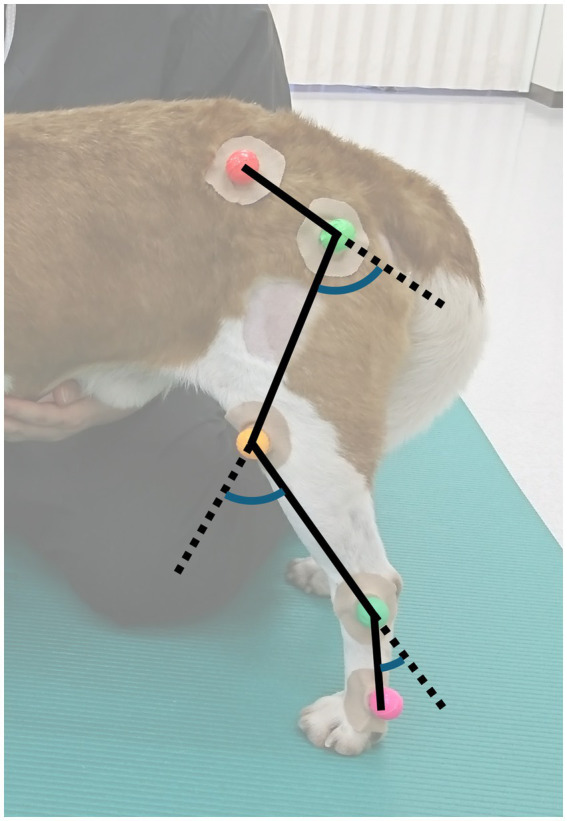
Marker placement and kinematic model for hindlimb analysis. Colored markers were placed on predefined anatomical landmarks: the iliac crest, the greater trochanter of the femur, the lateral femorotibial joint line (between the lateral femoral epicondyle and the fibular head), the lateral malleolus of the tibia, and the distal lateral aspect of the fifth metatarsal bone. Solid black lines connect adjacent markers to define the pelvic, femoral, tibial, and metatarsal segments, and the dashed lines illustrate the segment orientations used to compute the joint angles. Colored arcs indicate the hip, stifle, and tarsus joint angles, which were calculated as the difference between adjacent segment orientations and transformed as 180°–*θ* so that flexion corresponds to increasing values.

Colored markers were placed by a single examiner on predefined anatomical landmarks: the iliac crest, the greater trochanter of the femur, the femorotibial joint between the lateral epicondyle of the femur and the fibular head, the lateral malleolus of the tibia, and the distal lateral aspect of the fifth metatarsal bone (22, 24, 25). Solid lines connecting adjacent markers were used to define the pelvic, femoral, tibial, and metatarsal segments (Figure 1).

Joint angles were defined as the difference between the orientations of the adjacent limb segments at the hip (pelvis–femur), stifle (femur–tibia), and tarsus (tibia–metatarsus). To align the graphical representation with anatomical intuition, the measured angles (*θ*) were transformed as 180°–θ, such that physiological flexion corresponded to an increase in the angle value and extension to a decrease. This sign convention follows the common practice in human gait analysis, in which joint angle trajectories are typically defined such that flexion maps to increasing values and extension maps to decreasing values (4, 7). From a visualization standpoint, this convention yields cyclograms in which increasing angle values correspond to flexion plotted downward along the vertical axis and rightward along the horizontal axis, enhancing interpretability and facilitating comparisons across gait phases and between limbs.

#### 2.5.2 Definition of the gait phase

**Figure 2:**
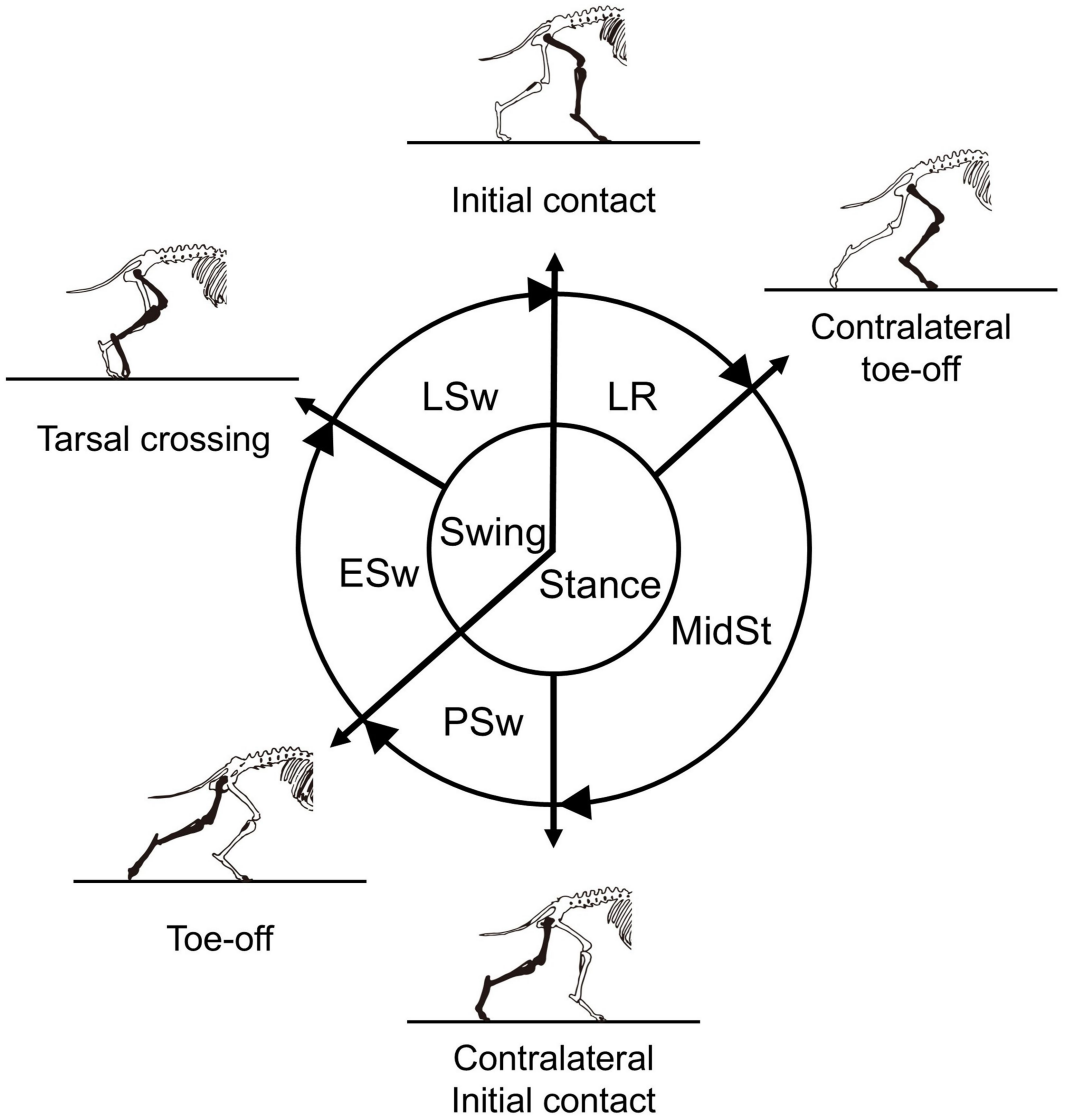
Schematic representation of canine hindlimb gait phase classification. The gait cycle is divided into stance and swing, which are further subdivided into the initial contact (IC) and five subsequent phases: loading response (LR), middle stance (MidSt), pre-swing (PSw), early swing (ESw), and late swing (LSw). Phase boundaries were defined by ipsilateral and contralateral initial contact and toe-off events, including contralateral toe-off, contralateral initial contact, ipsilateral toe-off, and tarsal crossing.

Based on our previous study (22), the canine gait cycle was subdivided into the initial contact (IC) and five subsequent phases: loading response (LR), middle stance (MidSt), pre-swing (PSw), early swing (ESw), and late swing (LSw) (Figure 2). IC was defined as the instant at which the hindlimb first contacted the floor. The LR extended from the IC until the contralateral hindlimb is lifted from the floor (contralateral toe-off). The MidSt extended from this event until the contralateral hindlimb contacted the floor again (contralateral IC). PSw extended from contralateral IC until the ipsilateral limb was lifted from the floor (ipsilateral toe-off). The ESw extended from the ipsilateral toe-off until the tarsal joint of the ipsilateral limb reached a position opposite the contralateral hindlimb in the sagittal plane, and the LSw extended from this event to the subsequent IC of the same limb (Figure 2).

#### 2.5.3 Preprocessing, stride normalization, and quality control

Marker coordinate trajectories (and digitized joint coordinates for the FHNO video data) were smoothed using a digital low-pass filter with an 8 Hz cut-off frequency (24). The hindlimb joint angles were calculated as described above. All video images were analyzed frame by frame by a single operator using the Kinema Tracer and ICpro systems.

For each valid gait cycle, the joint-angle trajectories were time-normalized to 0% to 100% of the gait cycle and were resampled to 101 points using linear interpolation to allow comparisons across trials and between dogs (24). After normalization, the joint angle data were first averaged across valid cycles within each dog and session, and the individual means were then averaged across dogs to obtain representative curves for the healthy cohort. In contrast, all geometric descriptors (A_abs and A_norm) were computed on a stride-wise basis prior to any averaging (Section 2.7).

### 2.6 Cyclogram construction and geometric descriptors

For each stride, the sagittal plane joint angle time series were time-normalized to 0%–100% of the gait cycle. Following the coordinate-level filtering and joint-angle computation described above, and to further reduce residual high-frequency fluctuations in stride-wise joint-angle time series, each joint-angle trajectory was smoothed using a cubic smoothing spline (R function smooth.spline; smoothing parameter spar = 0.6) and then resampled. The smoothed trajectories were resampled to *N* = 101 equally spaced points (including both endpoints) using linear interpolation. For numerical robustness in the subsequent noding and polygonization steps, the resampled joint-angle coordinates were rounded to 10^−3^ degrees (i.e., three decimal places) prior to geometric operations.

Planar angle–angle diagrams (cyclograms) were constructed for two adjacent joint pairs: hip–stifle and tarsus–stifle. For the hip–stifle pair, the hip angle was plotted on the *x*-axis and the stifle angle on the *y*-axis; for the tarsus–stifle pair, the tarsus angle was plotted on the *x*-axis and the stifle angle on the *y*-axis. Loop traversal followed the temporal progression of the gait cycle. Based on the gait-phase division illustrated in Figure 2, the stride trajectories were color-coded to indicate the five phases (excluding IC; LR, MidSt, PSw, ESw, and LSw), enabling the visual identification of phase-specific segments along the loop. The axis limits and aspect ratios were kept identical across panels and sessions to facilitate a direct visual comparison of the cyclogram morphology.

The primary geometric descriptor of inter-joint coordination was the absolute enclosed cyclogram area (A_abs, deg^2^). For each stride, the cyclogram trajectory in the angle–angle space was represented by an ordered set of points $(x_{i},y_{i}),i=1,\ldots ,N$ and the loop was closed by chord closure, that is, by setting $\left(x_{N+1},y_{N+1})=(x_{1},y_{1}\right)$ . Because cyclogram loops may self-intersect, the conventional algebraic polygon area computed by the shoelace formula (37) can be affected by cancellation between oppositely oriented regions (38).

Therefore, we defined A_abs as a polygonization-based total enclosed area for self-intersecting closed polylines. Specifically, we (i) applied a unary union operation to the chord-closed polyline to node all self-intersections (i.e., enforce that linework only meets at endpoints), and (ii) polygonized the resulting noded linework to obtain a set of bounded faces {P_j} .

A_abs was then defined as:

$$A_{\mathrm{abs}}=\sum_{j}\text{Area}\;(P_{j})$$

This definition yields a non-negative “total enclosed area” of the loop geometry that is robust to cancellation arising from self-intersections and can be implemented reproducibly using standard polygonization algorithms (39).

To reduce confounding from scale differences (e.g., breed/body size and acquisition modality), we also computed a dimensionless normalized area index:

$$A_{\mathrm{norm}}=\frac{A_{\mathrm{abs}}}{\mathrm{RO}M_{x}\times \mathrm{RO}M_{y}}$$

where *ROM_x* and *ROM_y* denote the stride-wise ranges of motion of the x-axis and y-axis joint angles within the stride.

Although A_norm reduces scale-related effects by expressing A_abs relative to the stride-wise joint excursions (*ROM_x and ROM_y*), ROM limitation itself can be part of the pathological gait phenotype. Therefore, A_norm is reported only as an exploratory, dimensionless adjunct to A_abs and is not used as a stand-alone basis for pathology-specific inference in this study.

All computations were performed using custom scripts in R software (version 4.3.2; R Foundation for Statistical Computing, Vienna, Austria). Polygonization and unary union operations were implemented via GEOS-based simple-feature operations in R (sf ecosystem) (39).

For the healthy cohort, cyclograms were used descriptively to illustrate canonical loop morphology and inter-joint coordination. In addition, stride-wise A_abs and A_norm values were computed to quantify within-session repeatability in the healthy cohort (Section 2.7.1). Quantitative A_abs values were not used for numerical between-sample comparisons between the healthy reference sample and the single FHNO case because differences in breed, body size, walking condition, and recording modality between the healthy beagles and the clinical FHNO case would confound interpretation. In particular, prior canine studies have shown that treadmill versus overground ambulation can yield broadly similar waveform shapes while still producing analysis-dependent differences (20), and breed/conformation can influence hindlimb kinematics, with hip joint kinematics differing between breeds even under controlled treadmill walking (40). Moreover, the healthy cohort was recorded using a three-dimensional skin-marker-based model, whereas the FHNO case was analyzed using a two-dimensional sagittal-plane video approach, which may further affect estimated joint angles and cyclogram geometry. For the FHNO case, A_abs was computed for each stride, joint pair, and session and served as the primary quantitative outcome.

### 2.7 Outcomes and statistical analysis

#### 2.7.1 Healthy reference cohort: repeatability and descriptive statistics

For each healthy beagle, 10 valid gait cycles were analyzed. For each cycle and joint pair (hip–stifle and tarsus–stifle), we computed stride-wise A_abs (deg^2^) and A_norm (dimensionless) as described in Section 2.6. To demonstrate cohort consistency, we report dog-level mean ± SD across cycles for each joint pair and summarize across dogs using the mean ± SD of dog-level means (Results, Section 3.1; Tables 1, 2).

**Table 1:** Repeatability and measurement error of A_abs in healthy beagles.

Metric	hip–stifle	tarsus–stifle
*n* (dogs)	8	8
*n* (cycles per dog)	10	10
A_abs (deg^2^), mean ± SD	813.18 ± 114.88	619.88 ± 129.00
ICC(1,1) (95% CI)	0.59 (0.24–0.80)	0.67 (0.23–0.89)
ICC(1,10) (95% CI)	0.93 (0.76–0.98)	0.95 (0.74–0.99)
SEM / MDC95 (deg^2^)	93.04/257.90	89.24/247.37
SEM_10 / MDC95_10 (deg^2^)	29.42/81.55	28.22/78.23

Values are reported for each joint-pair cyclogram (hip–stifle, tarsus–stifle) based on *n* = 8 healthy beagles with 10 consecutive gait cycles per dog. A_abs mean ± SD denotes the mean ± SD across dog-level means, where each dog-level mean was computed from 10 cycles. A_abs was computed using the polygonization-based total enclosed area definition. Within-session repeatability was quantified using a one-way random-effects intraclass correlation coefficient [ICC (1,1)] and the corresponding average-measures ICC(1,10), treating dog as the random effect and cycle as the repeated measure. 95% confidence intervals (CI) for ICCs are bootstrap percentile intervals obtained by resampling dogs with replacement while retaining the within-dog 10-cycle structure (B = 5,000). Measurement error was summarized using the standard error of measurement (SEM, deg^2^) and the minimal detectable change at the 95% confidence level (MDC95, deg^2^; MDC95 = 1.96 × √2 × SEM). For the mean of 10 consecutive cycles, SEM_10 = SEM/√10 and MDC95_10 = 1.96 × √2 × SEM_10. A_abs, absolute enclosed cyclogram area; SD, standard deviation; ICC, intraclass correlation coefficient; CI, confidence interval; SEM, standard error of measurement; MDC95, minimal detectable change at the 95% confidence level; SEM_10, standard error of measurement for the mean of 10 consecutive cycles; MDC95_10, minimal detectable change at the 95% confidence level for the mean of 10 consecutive cycles.

**Table 2:** Individual dog-level descriptive statistics for cyclogram area (A_abs) in healthy beagles.

Pair	Dog ID	*n* (strides per session)	A_abs (deg^2^), mean ± SD	CV (%)	A_norm, mean
hip–stifle	A	10	835.16 ± 65.13	7.80	0.64
hip–stifle	B	10	636.39 ± 40.48	6.36	0.55
hip–stifle	C	10	754.76 ± 141.10	18.69	0.51
hip–stifle	D	10	706.20 ± 57.99	8.21	0.53
hip–stifle	E	10	799.60 ± 45.57	5.70	0.58
hip–stifle	F	10	974.27 ± 159.84	16.41	0.54
hip–stifle	G	10	850.88 ± 90.18	10.60	0.58
hip–stifle	H	10	948.21 ± 65.91	6.95	0.59
tarsus–stifle	A	10	539.35 ± 24.89	4.61	0.39
tarsus–stifle	B	10	415.91 ± 26.40	6.35	0.40
tarsus–stifle	C	10	547.34 ± 103.46	18.90	0.41
tarsus–stifle	D	10	777.27 ± 67.59	8.70	0.41
tarsus–stifle	E	10	554.38 ± 34.67	6.25	0.43
tarsus–stifle	F	10	637.78 ± 182.29	28.58	0.34
tarsus–stifle	G	10	703.60 ± 94.73	13.46	0.43
tarsus–stifle	H	10	783.43 ± 61.01	7.79	0.43

Dog IDs (A–H) denote the eight healthy beagles. For each dog and joint-pair cyclogram (hip–stifle, tarsus–stifle), values summarize n = 10 consecutive gait strides within a single session. A_abs (deg^2^), mean ± SD represents the dog-level mean and within-dog SD computed across the 10 gait cycles. CV (%) denotes within-dog dispersion, calculated as 100 × (SD/mean). A_norm is a dimensionless ROM-normalized index [A_abs / (ROM_x × ROM_y)] reported as an exploratory adjunct (see “Section 2.6”). These dog-level summaries support interpretation of within-session repeatability estimates reported in Table 1 (see “Section 2.7.1”).

Within-session repeatability across dogs was quantified using a one-way random-effects intraclass correlation coefficient (ICC (1,1)) and the corresponding average-measures ICC (1,10), treating dog as the random effect and cycle as repeated measure (41).

Measurement error was summarized using the standard error of measurement (SEM) and the minimal detectable change at the 95% confidence level (MDC95 = 1.96 × √2 × SEM) (42).

To provide uncertainty intervals for ICC estimates under the small-sample setting (*n* = 8 dogs), we derived 95% bootstrap percentile intervals (B = 5,000) by resampling dogs with replacement and retaining the original 10-cycle within-dog structure in each bootstrap replicate.

#### 2.7.2 FHNO longitudinal analysis

For the FHNO case, stride-level (gait-cycle-level) A_abs values were computed for each joint pair (hip–stifle and tarsus–stifle) at POD14, POD21, and POD31, with *n* = 10 strides per session. Longitudinal changes were evaluated descriptively by comparing A_abs across sessions within each joint pair and estimating the difference in session-wise mean A_abs for prespecified contrasts (POD21–POD14, POD31–POD14, POD31–POD21).

All analyses were conducted in R. For each joint pair and session, the session-wise mean A_abs was summarized with a 95% bootstrap confidence interval (CI) (percentile method; B = 5,000) based on nonparametric resampling of strides with replacement; these session-wise summaries (mean, SD, CV, and 95% bootstrap CI) are reported in Table 3. For each pre-specified longitudinal contrast, strides were resampled with replacement separately within each session, and the difference in bootstrap means (ΔA_abs) was calculated for each bootstrap replicate; a 95% bootstrap CI (percentile method) was then derived from the resulting ΔA_abs distribution; these between-day differences (ΔA_abs) and their bootstrap CIs are reported in Table 4. Given the n-of-1 design, these bootstrap intervals are reported as descriptive uncertainty summaries rather than formal hypothesis tests. Within-session dispersion was calculated using the coefficient of variation (CV, %) = 100 × (standard deviation (SD)/mean).

**Table 3:** Stride-wise absolute cyclogram area (A_abs) of the operated hindlimb (FHNO side) at each postoperative day for the hip–stifle and tarsus–stifle joint pairs.

Pair	Day	*n* (strides per session)	Mean A_abs (deg^2^)	SD (deg^2^)	95% bootstrap CI (deg^2^)	CV (%)
Hip–stifle	POD14	10	112.24	74.93	71.7–157.82	66.76
Hip–stifle	POD21	10	372.74	237.4	244.1–513.8	63.69
Hip–stifle	POD31	10	395.09	120.93	322.1–468.2	30.61
Tarsus–stifle	POD14	10	185.73	143.6	116.6–278.7	77.32
Tarsus–stifle	POD21	10	505.97	315.22	340.0–700.1	62.3
Tarsus–stifle	POD31	10	543.78	229.36	406.2–677.1	42.18

For each joint pair and postoperative day (POD14, POD21, POD31), *n* = 10 valid strides of the operated hindlimb were analyzed. Values are reported as the mean A_abs (deg^2^), standard deviation (SD, deg^2^), coefficient of variation (CV, %), and the 95% bootstrap percentile confidence interval (CI, deg^2^) for the mean (B = 5,000; resampling strides with replacement). A_abs was computed using the polygonization-based total enclosed area method described in “Section 2.6.” A_abs, absolute enclosed cyclogram area; FHNO, femoral head and neck ostectomy; POD, postoperative day; SD, standard deviation; CV, coefficient of variation; CI, confidence interval.

**Table 4:** Bootstrap estimates of between-day differences in mean absolute cyclogram area (ΔA_abs, deg^2^) for the operated hindlimb (FHNO side).

Pair	Contrast	Mean ΔA_abs (deg^2^)	95% bootstrap CI (deg^2^)
Hip–stifle	POD21 − POD14	260.50	124.15–409.07
Hip–stifle	POD31 − POD14	282.85	199.16–364.83
Hip–stifle	POD31 − POD21	22.34	−132.9–175.47
Tarsus–stifle	POD21 − POD14	320.24	128.97–536.17
Tarsus–stifle	POD31 − POD14	358.06	200.82–508.81
Tarsus–stifle	POD31 − POD21	37.81	−195.84–257.26

For each joint pair (hip–stifle, tarsus–stifle) and postoperative contrast (POD21–POD14, POD31–POD14, POD31–POD21), the between-day difference in the session-wise mean A_abs (ΔA_abs) and its 95% bootstrap percentile confidence interval (CI) are reported. Differences were calculated as later POD minus earlier POD; therefore, positive values indicate a larger mean A_abs on the later postoperative day. Bootstrap CIs were obtained by nonparametric resampling of strides with replacement within each postoperative day (B = 5,000; *n* = 10 strides per day) as described in “Section 2.7.2.” A_abs was computed using the polygonization-based total enclosed area (Route A) described in “Section 2.6.” A_abs, absolute enclosed cyclogram area; ΔA_abs, between-day difference in mean A_abs; FHNO, femoral head and neck ostectomy; POD, postoperative day; CI, confidence interval.

#### 2.7.3 Exploratory discrimination analysis

To explore whether the cyclogram-based indices have the potential to discriminate a single clinical case from a small healthy reference sample, we performed an exploratory single-case vs. controls comparison using the modified *t*-test proposed by Crawford and Howell, applied to dog-level healthy summary values (A_abs) (43). For the healthy reference sample, dog-level mean A_abs values were computed across the 10 consecutive cycles for each beagle (*n* = 8 dogs). For the FHNO case, the session-level mean A_abs at each postoperative time point (POD14, POD21, POD31) was compared against the healthy reference distribution using two-sided Crawford–Howell tests (df = 7), and t statistics and *p* values are reported as exploratory indicators of deviation from the healthy reference sample. Because of substantial between-group heterogeneity (breed/body size, treadmill vs. overground, 3D vs. 2D), these analyses were interpreted cautiously and were not used to draw pathology-specific conclusions.

## 3 Results

### 3.1 Canonical hindlimb cyclograms in healthy beagles

**Figure 3:**
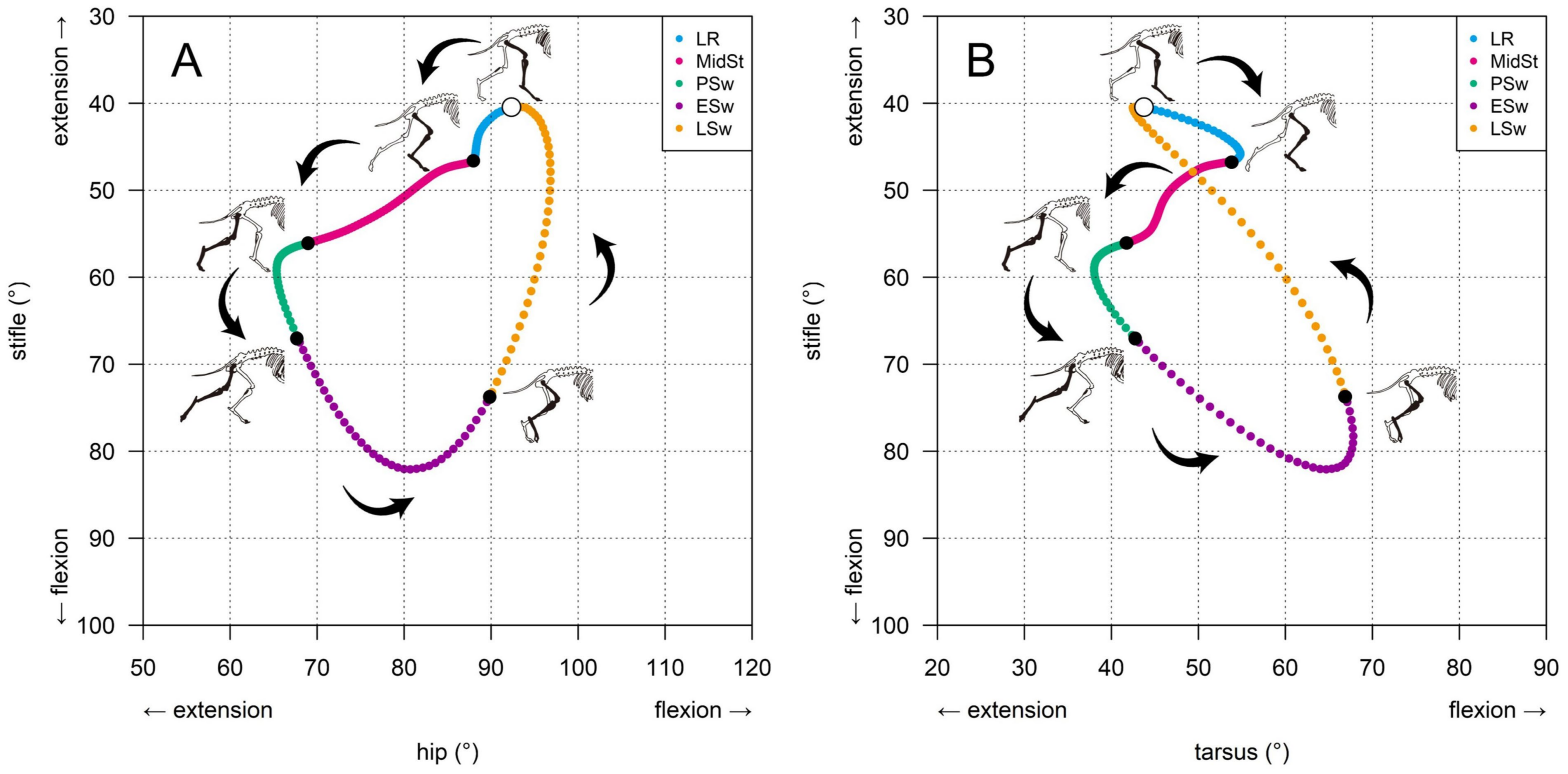
Canonical hindlimb inter-joint cyclograms in healthy beagle dogs during treadmill walking. **(A)** Hip–stifle angle–angle diagram. **(B)** Tarsus–stifle angle–angle diagram. In each panel, stifle angle (vertical axis) is plotted against hip or tarsus angle (horizontal axis), with increasing values corresponding to greater flexion (downward and rightward, respectively). The colored curve represents the mean cyclogram across strides and eight clinically healthy Beagles. Arrows indicate the direction of progression through the gait cycle. An open circle on each loop marks the timing of initial contact (IC). Colored segments denote gait sub-phases: loading response (LR, blue), middle stance (MidSt, pink), pre-swing (PSw, green), early swing (ESw, purple), and late swing (LSw, orange). Silhouetted dog outlines illustrate representative limb configurations at selected points along each loop.

In healthy beagle dogs, both hip–stifle and tarsus–stifle cyclograms formed smooth closed trajectories (Figure 3). The hip–stifle cyclogram described a broad counterclockwise loop with a slightly inverted-triangular, elliptical outline (Figure 3A). For clarity, gait phases are described here using full terms (see Figure 3 for phase-specific color coding). During stance, the loading response and middle stance portions of the loop occupied the upper region, where the stifle gradually moved from extension toward flexion, whereas the hip progressed from moderate flexion toward extension. From pre-swing to early swing, the loop descended toward more flexed stifle angles, as both joints flexed in parallel. During late swing, the loop ascended back toward the region of initial contact, capturing the re-extension of the stifle accompanied by renewed hip flexion immediately before the subsequent initial contact.

The tarsus–stifle cyclogram exhibited a figure-of-eight pattern (Figure 3B). During loading response, the combined flexion of the tarsus and stifle formed the upper lobe of a clockwise figure-of-eight trajectory. From middle stance onward, the trajectory reversed and became counter-clockwise. During the transition from loading response to middle stance, the tarsus switched from flexion to extension, whereas the stifle remained in a sustained flexed position. In pre-swing, as the limb approached toe-off, the tarsus transitioned from extension to flexion, whereas the stifle continued to flex. In early swing, both joints flexed further; thereafter, as the tarsus and the stifle switched to extension toward the subsequent initial contact, the trajectory curved toward the initial contact. In late swing, similar to the terminal portion of early swing, both joints remained in extension until the subsequent initial contact.

Descriptive statistics and repeatability of A_abs in healthy beagles are summarized in Tables 1, 2. Using the Route A polygonization-based definition, the mean A_abs (mean ± SD across dog-level means) was 813.2 ± 114.9 deg^2^ for the hip–stifle cyclogram and 619.9 ± 129.0 deg^2^ for the tarsus–stifle cyclogram. Within-session repeatability across dogs was moderate to good: ICC (1,1) was 0.59 (95% bootstrap CI: 0.24–0.80) for hip–stifle and 0.67 (0.23–0.89) for tarsus–stifle; average-measures ICC (1,10) was 0.93 (0.76–0.98) and 0.95 (0.74–0.99), respectively (Table 1). The corresponding SEM and MDC95 values were 93.0 and 257.9 deg^2^ (hip–stifle) and 89.2 and 247.4 deg^2^ (tarsus–stifle) (Table 1). Individual dog-level means and within-dog SD/CV across cycles are reported in Table 2.

### 3.2 Qualitative evolution of cyclogram morphology after FHNO

**Figure 4:**
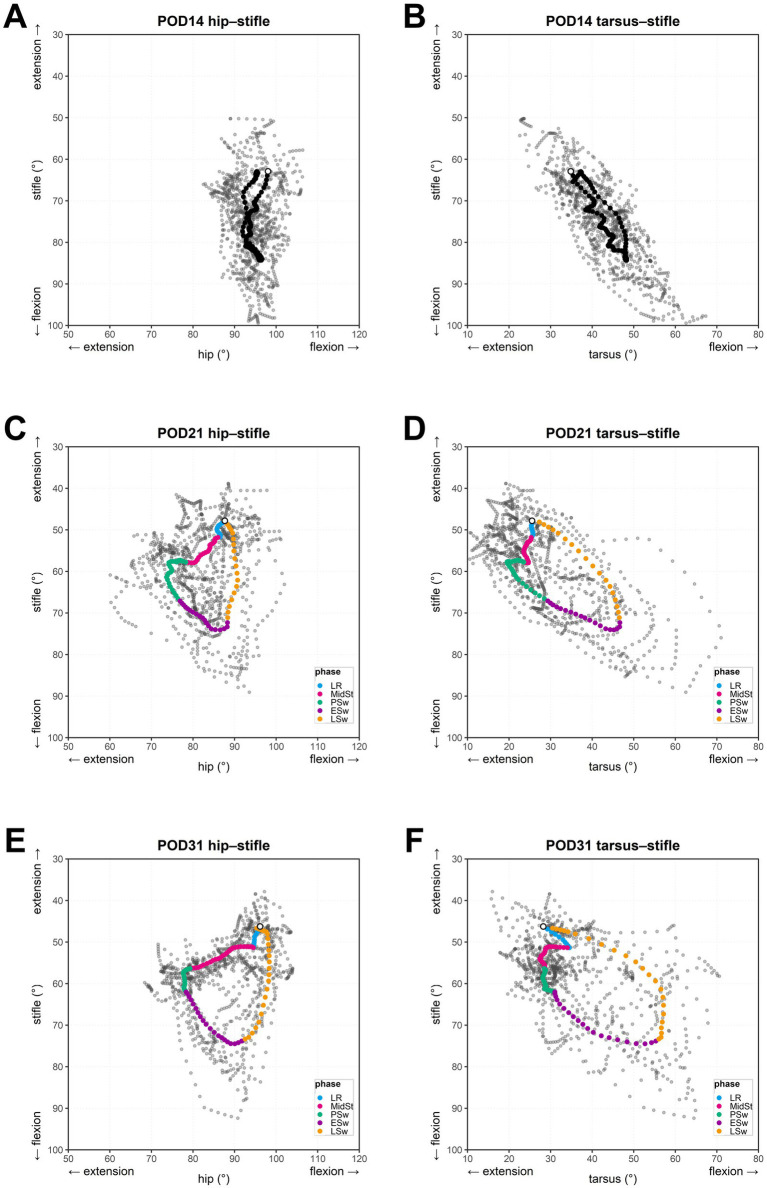
Longitudinal evolution of hip–stifle and tarsus–stifle cyclograms in the operated hindlimb. Panels **(A–F)** show cyclograms of the operated hindlimb on postoperative days (POD) 14 **(A,B)**, 21 **(C,D)**, and 31 **(E,F)**. On each day, the left and right panels correspond to the hip–stifle and tarsus–stifle joint pairs, respectively. In each panel, the gray points represent all the time-normalized angle-angle points from all the strides recorded during that session, and the bold loop depicts the session-averaged trajectory, obtained by averaging the time-normalized joint-angle trajectories across strides. At POD14, the gait phases could not be reliably identified; therefore, the session-averaged loop is shown as a single non-phase-segmented black trajectory. At POD21 and POD31, the gait phases were classifiable, and the representative loop was color-segmented according to the phase (LR, MidSt, PSw, ESw, LSw). An open circle on each loop indicates the timing of the initial contact (IC). Axes indicate joint angles (degrees) for the hip or tarsus (x-axis) and the stifle (y-axis); as described in the Methods section, flexion increases to the right and downward, and extension increases to the left and upward.

In the FHNO case, hindlimb kinematics on POD14 did not allow reliable identification of individual gait phases; therefore, gait-phase classification was not applied. Consequently, the POD14 cyclograms shown in Figures 4A,B are presented without phase-specific color coding and exhibit a markedly restricted loop morphology. By POD21 and POD31, however, gait phases could be clearly identified, and the cyclogram loops progressively expanded (Figures 4C–F).

At POD14, the hip–stifle cyclogram had collapsed into a narrow, almost vertically oriented band, indicating markedly reduced hip excursion with motion occurring predominantly at the stifle (Figure 4A). The tarsus–stifle cyclogram on POD14 showed a similarly constricted, vertically aligned trajectory with minimal tarsal excursion, with most of the variation occurring at the stifle (Figure 4B). In each panel, the gray points represent all strides recorded for the operated limb on the indicated POD, and the bold loop (black on POD14 and color-segmented on POD21 and POD31) depicts the session-averaged trajectory obtained by averaging the time-normalized joint-angle trajectories across strides, which is shown for phase-specific visualization.

At POD21, the cyclogram loops for both joint pairs were enlarged compared with POD14, with widening along the hip and tarsus (x-) axis (Figures 4C,D). In the hip–stifle diagram, an inverted-triangular outline emerged; however, the phase-specific trajectories remained irregular and jagged. The tarsus–stifle loop likewise expanded and approached an elliptical shape, but the tarsal angles were still biased toward extension with limited tarsal flexion during loading response. The upper lobe of the figure-of-eight pattern observed in healthy beagle dogs (Figure 3B) was not evident.

At POD31, the overall shapes of both hip–stifle and tarsus–stifle cyclograms further broadened along the hip and tarsus (x-) axes, respectively (Figures 4E,F). Irregular phase-specific trajectories on POD21 appeared smoother and more continuous. In the hip–stifle diagram, stance-phase (loading response, middle stance, pre-swing) segments showed less pronounced stifle flexion, and the excursion of hip extension was increased, particularly during middle stance. The swing-phase segments (early swing and late swing) formed a fuller and smoother loop. In the tarsus–stifle loop, the range of tarsal flexion was greater than that observed on POD21, resulting in a wider spread along the x-axis. Although tarsal flexion was evident during loading response, the upper lobe of the figure-of-eight pattern, characteristic of healthy beagle dogs (Figure 3B), was not apparent.

### 3.3 Quantitative changes in cyclogram area and stride-to-stride variability

#### 3.3.1 Hip–stifle cyclogram area

For the hip–stifle joint pair, stride-level cyclogram area (A_abs) of the operated limb increased over time, consistent with the visually apparent loop enlargement described above (Table 3). The mean A_abs values (95% bootstrap CI) were 112.2 deg^2^ (71.7–157.8) on POD14, 372.7 deg^2^ (244.1–513.8) on POD21, and 395.1 deg^2^ (322.1–468.2) on POD31. These estimates are descriptive for this single case and are presented to illustrate within-dog longitudinal tracking.

Bootstrap estimates of between-day differences in A_abs are summarized in Table 4. The mean difference for POD21–POD14 was +260.5 deg^2^ (95% CI 124.2–409.1). The mean difference for POD31–POD14 was +282.8 deg^2^ (199.2–364.8), whereas POD31–POD21 was +22.3 deg^2^ (−132.9–175.5).

#### 3.3.2 Tarsus–stifle cyclogram area

In the tarsus–stifle pair, the stride-level A_abs values showed a temporal pattern broadly similar to that observed for the hip–stifle pair, with a tendency to increase over time (Table 3). Mean A_abs values were 185.7 deg^2^ (95% CI: 116.6–278.7) at POD14, 506.0 deg^2^ (340.0–700.1) at POD21, and 543.8 deg^2^ (406.2–677.1) at POD31.

Bootstrap estimates of between-time-point mean differences in A_abs for the tarsus–stifle pair are summarized in Table 4. The mean differences for POD21–POD14 and POD31–POD14 were +320.2 deg^2^ (129.0–536.2) and +358.1 deg^2^ (200.8–508.8), respectively. In contrast, the mean difference for POD31–POD21 was +37.8 deg^2^ (−195.8–257.3).

#### 3.3.3 Stride-to-stride variability

The stride-to-stride variability in A_abs is summarized by the CV reported in Table 3. For the hip–stifle pair, the CV was 66.8% on POD14, 63.7% on POD21, and 30.6% on POD31. For the tarsus–stifle pair, the corresponding CV values were 77.3, 62.3, and 42.2%.

#### 3.3.4 Exploratory single-case vs. controls comparison

Exploratory single-case vs. controls comparisons (Crawford–Howell modified t-test; two-sided; df = 7), using dog-level healthy mean A_abs values (*n* = 8) as the reference distribution, suggested that the FHNO case had lower hip–stifle A_abs than the healthy reference distribution at POD14 (*t* = −5.75, *p* = 0.0007), POD21 (*t* = −3.61, *p* = 0.0086), and POD31 (*t* = −3.43, *p* = 0.0110), whereas for the tarsus–stifle pair the FHNO case was lower only at POD14 (*t* = −3.17, *p* = 0.0156) and not at POD21 (*t* = −0.83, *p* = 0.4326) or POD31 (*t* = −0.56, *p* = 0.5954). Given the pronounced between-sample heterogeneity (breed/body size, treadmill vs. overground, 3D vs. 2D), these results are reported as exploratory indicators of deviation from the healthy reference sample and are not interpreted as pathology-specific inference.

## 4 Discussion

In this study, we used planar hip–stifle and tarsus–stifle cyclograms of the canine hindlimb to (i) characterize representative inter-joint coordination patterns in healthy beagle dogs and (ii) evaluate, in a unilateral FHNO case, the extent to which the absolute enclosed cyclogram area (A_abs) and its within-session stride-to-stride variability can descriptively capture early postoperative changes. Taken together, our findings indicate that cyclograms compactly represent how the canine hindlimb joints coordinate throughout the gait cycle and that A_abs may serve as a simple quantitative metric for tracking rehabilitation progress over time within an individual dog under consistent acquisition/analysis conditions.

### 4.1 Cyclogram characteristics of healthy beagle dogs

In clinically healthy beagle dogs, both hip–stifle and tarsus–stifle cyclograms formed smooth, closed trajectories, indicating a stable pattern of hindlimb inter-joint coordination during treadmill walking at a comfortable speed. The hip–stifle cyclogram described a broad, counterclockwise loop with a slightly inverted-triangular, elliptical outline, reflecting phase-shifted flexion–extension of the hip and stifle across stance and swing. In contrast, the tarsus–stifle cyclogram exhibited a characteristic figure-of-eight configuration, suggesting more complex timing relationships between the tarsus and stifle motions. To complement these canonical qualitative patterns, we quantified stride-wise cyclogram area indices in the healthy cohort and demonstrated within-session repeatability and measurement error estimates (Tables 1, 2), thereby supporting the methodological consistency of the proposed approach under standardized treadmill conditions.

Although we present only cyclograms, the directions in which each loop progressed and the locations of the stance and swing phases along the loops were consistent with the hip, stifle, and tarsus flexion–extension waveforms reported in previous sagittal plane angle–time kinematic studies of hindlimb motion in healthy dogs during steady walking (19, 20, 25–27, 44). Therefore, even without separately presenting joint angle–time curves, these canonical cyclograms can be interpreted as a concise graphical summary of the sagittal-plane hindlimb kinematics in dogs. However, because the cyclograms were derived from only eight clinically healthy beagles walking on a treadmill at a single comfortable speed, they should be regarded as preliminary breed- and condition-specific reference patterns rather than universally normative data for all canine hindlimbs. To the best of our knowledge, this study is among the first to present canine hindlimb cyclograms, extending previous joint-by-joint kinematic descriptions into a framework that directly represents inter-joint coordination. In addition, because self-intersections can occur in canine cyclograms (e.g., the figure-of-eight tarsus–stifle loops), our area computation adopted a polygonization-based total enclosed area definition (A_abs) that is robust to cancellation from oppositely oriented regions (Section 2.6).

Although the detailed loop geometry in dogs naturally differs from that reported in humans, our interpretation of these cyclograms is conceptually consistent with classic human hip–knee cyclogram studies (6, 7) and more recent work on knee osteoarthritis (9). Specifically, our findings in dogs support the notion that loop shape, orientation, and the presence or absence of self-intersections can serve as compact descriptors of when and to what extent each joint moves throughout the gait cycle. Accordingly, the primary contribution of the present work is the methodological characterization and repeatability assessment in healthy dogs, which provides a quantitative basis for subsequent clinical applications.

Furthermore, in the present study, we interpreted cyclogram trajectories within a previously developed and validated five-sub-phase canine hindlimb gait model (22). By mapping the five gait sub-phases onto the cyclogram loops, we were able to localize deviations in inter-joint coordination to specific sub-phases, rather than describing abnormalities only in terms of generic stance or swing-phase lameness. This framework provides a basis for future studies that integrate cyclogram features with kinetic and electromyographic data, thereby enabling more mechanistic analyses of which joint and sub-phase of the gait cycle primarily contribute to particular lameness patterns or rehabilitation responses.

The canonical loops derived here in beagles are breed-specific by design, but they nevertheless offer a practical visual reference against which diseased or post-treatment hindlimb coordination patterns may be qualitatively compared. These results also provide a basis for future investigations to examine whether similar loop geometries and sub-phase-specific coordination patterns are preserved across different canine breeds. Where cross-breed or cross-modality comparisons are desired, normalization strategies (e.g., ROM-normalized indices) should be evaluated under methodologically consistent conditions (see “Limitations”).

### 4.2 Cyclogram changes in the FHNO case

In the FHNO case, the cyclogram morphology appeared to reflect both the early postoperative disruption and the subsequent partial recovery of inter-joint coordination. On POD14, the loops of both joint pairs were narrow in the horizontal direction and appeared to be vertically elongated. In the hip–stifle cyclogram, the stifle moved through a relatively wide angular range, whereas hip excursion was markedly restricted, which may reflect a protective gait strategy in the early postoperative period (e.g., postoperative pain, reduced weight bearing, and/or altered mechanics at the operated hip).

In the tarsus–stifle cyclogram, the characteristic figure-of-eight pattern observed in healthy beagles disappeared, and the loop was dominated by stifle motion. This configuration may be compatible with reduced shock absorption at the tarsus, which is normally supported by controlled tarsal flexion and energy absorption by the tarsal extensors after IC in dogs (23). In this context, the altered cyclogram can be used to visualize disrupted inter-joint coordination associated with impaired tarsal function in the early postoperative period. From a clinical perspective, in the early period after FHNO, dogs often avoid loading the operated hindlimb and exhibit limited hip extension. These functional limitations are consistent with previous outcome studies after femoral head and neck excision/ostectomy that document persistent postoperative gait impairment and reduced use of the operated limb, particularly in the early postoperative period (45). Kinematic gait analysis after femoral head and neck ostectomy has further suggested that reduced coxofemoral excursion/reluctance to extend the hip may be accompanied by compensatory strategies at more distal joints, with decreased hip amplitude being compensated mainly via increased extension of the tarsal joint (46). Collectively, such compensatory patterns may contribute to a stiffer-appearing gait during early recovery.

As time progressed to POD21 and POD31, the loops for both joint pairs gradually expanded outward and, although not fully normalized, became visually more similar to the canonical patterns of healthy beagles. The hip–stifle loop became broader, indicating that hip motion had begun to re-engage more fully throughout the gait cycle. In the tarsus–stifle loop, the mediolateral width also increased, and the trajectory began to suggest the re-emergence of a figure-of-eight configuration; however, the upper lobe typical of healthy beagles remained underdeveloped by POD31. Quantitative summaries of stride-level A_abs and longitudinal contrasts are reported as descriptive estimates (Results; Tables 3, 4) and were not used for formal between-cohort inference. We emphasize that the FHNO component is included to demonstrate feasibility of longitudinal within-dog monitoring using cyclogram-derived metrics under clinical acquisition constraints, not to support pathology-specific between-sample inference or claims of clinical classification performance. More broadly, the incomplete restoration pattern was consistent with the persistent functional deficits described in long-term follow-up studies after FHNO, in which residual limitations in hip extension, muscle atrophy, and asymmetric weight bearing are frequently reported despite substantial improvements in pain and favorable owner-reported outcomes (29–32). Previous kinetic (force-plate) studies have quantified persistent alterations in limb loading after femoral head and neck excision arthroplasty using ground reaction force variables (32). Such objective asymmetries may persist despite clinically acceptable outcomes reported in follow-up studies after femoral head and neck excision/ostectomy (45). Kinematic studies, in contrast, typically report joint-specific angle–time waveforms and discrete measures such as range of motion, describing how individual joints change during recovery (30). Cyclogram analysis complements these conventional approaches by explicitly characterizing inter-joint coordination as a coupled trajectory (joint–joint relationship), thereby providing an interpretable summary of how two joints move together rather than separately. In the present FHNO case, both hip–stifle and tarsus–stifle cyclograms showed progressive changes in loop geometry from POD14 to POD31, accompanied by an increase in mean A_abs and a reduction in stride-to-stride variability, suggesting a transition toward larger and more stable coordination patterns during early recovery. Accordingly, cyclogram-derived geometric features such as A_abs may offer a practical joint-pair–specific index for longitudinal monitoring of postoperative coordination patterns and compensatory coupling across joints that may be less apparent when inspecting single-joint angle–time curves alone.

### 4.3 Role and interpretation of A_abs

In human gait analysis, cyclogram-based metrics such as enclosed area, perimeter, and loop-shaped descriptors have been used to quantify inter-joint coordination and differentiate between patient groups (6–9, 12–14). Recent studies have shown that cyclogram-derived indices, including enclosed areas and coordination indices based on loop geometry, are sensitive to disease progression and surgical status in conditions such as adolescent idiopathic scoliosis and knee osteoarthritis (12–14). In this context, using A_abs as a summary metric in dogs is conceptually consistent with current human gait analysis practices.

In the present work, A_abs was defined as a polygonization-based “total enclosed area” for chord-closed cyclogram loops (“Section 2.6”), enabling a cancellation-robust area definition even when loops self-intersect. A_abs provides a single scalar measure of loop size and thus serves as a compact quantitative descriptor of inter-joint coordination and gait pattern. Importantly, we quantified within-session repeatability and measurement error for A_abs in healthy beagles (Results; Tables 1, 2), showing moderate-to-good single-cycle repeatability and excellent repeatability when computed from an average across 10 consecutive cycles, alongside SEM and MDC95 values that can guide interpretation of longitudinal change under standardized conditions. In the illustrative FHNO case, the mean A_abs values increased from POD14 to POD21 and POD31 for both hip–stifle and tarsus–stifle joint pairs (Results; Table 3), and bootstrap-based estimates of between-time-point differences are shown in Table 4. Because this component is n-of-1, these intervals are reported as descriptive uncertainty summaries rather than hypothesis tests, and they should be interpreted as demonstrating feasibility of within-dog tracking rather than supporting generalizable treatment effects.

Previous kinetic studies have shown that the peak vertical force and vertical impulse remain reduced in the operated limb for months to years, indicating persistent asymmetrical weight bearing despite satisfactory clinical recovery (29, 30, 32). In this context, our finding that A_abs remained relatively low on the operated limb during early rehabilitation suggests that inter-segmental coordination deficits coexist with these kinetic asymmetries and represent a complementary aspect of locomotor impairment that ground reaction force measurements alone may not detect. Therefore, cyclogram-based metrics may help explain some variability in functional outcomes after FHNO and support the combined use of kinematic and kinetic analyses in future rehabilitation studies.

A critical point, however, is that larger A_abs is not inherently “better” or “worse” in a universal sense. In human knee osteoarthritis, the enclosed hip–knee cyclogram area is larger than that in healthy controls and has been interpreted as an indicator of abnormal joint coupling and disease severity (8, 9). In the FHNO case described herein, early postoperative cyclograms appeared, on visual inspection, geometrically smaller and more horizontally compressed than the canonical loops from healthy beagles and then expanded toward a configuration that qualitatively resembled this reference pattern over time. Accordingly, the interpretation of A_abs should remain context-dependent and should be anchored to an appropriate reference pattern (joint pair, breed, and acquisition modality), particularly when comparing across heterogeneous datasets.

To address scale-related confounding (e.g., breed/body size and acquisition modality), we additionally computed a ROM-normalized index [A_norm = A_abs/(ROM_x × ROM_y)]. However, given the pronounced between-group heterogeneity (treadmill vs. overground; 3D vs. 2D; Beagle vs. Toy Poodle), A_norm is reported only as an exploratory normalization and was not used to draw pathology-specific conclusions in this study. Because ROM restriction itself can be clinically meaningful, A_norm is interpreted only as an exploratory adjunct and does not replace A_abs when describing within-dog postoperative patterns.

In addition, we report an exploratory single-case vs. controls comparison (Crawford–Howell modified *t*-test) using dog-level healthy A_abs summary values as a reference distribution. These results are presented only as descriptive indicators of deviation under the study conditions and are not interpreted as evidence of pathology-specific effects or clinical classification performance given the pronounced between-sample heterogeneity.

Notably, the recovery trajectories of A_abs differed between the two joint pairs. In the tarsus–stifle pair, A_abs increased relatively early after surgery (from POD14 to POD21) and then changed modestly thereafter, whereas in the hip–stifle pair, A_abs continued to increase over a longer period from POD14 to POD31. This observation is presented descriptively for the single case and should be tested in larger cohorts before being interpreted mechanistically.

Overall, the healthy-cohort repeatability metrics (ICC, SEM, and MDC95) provide the primary validation foundation for interpreting cyclogram-derived indices, whereas the FHNO case and the associated exploratory discrimination results are presented primarily to illustrate feasibility of within-dog monitoring and to motivate future studies under methodologically harmonized conditions.

### 4.4 Stride-to-stride variability and stabilization of coordination patterns

The stride-to-stride variability of A_abs, expressed as CV, decreased over time in both joint pairs (Table 3). In this illustrative case, the stride-level A_abs values fluctuated widely within the same session, indicating an unstable gait pattern. As recovery progressed, the cyclogram loops not only increased in size but also became more similar in shape across strides, suggesting that a more consistent movement pattern was re-established. Because the FHNO component is n-of-1, these changes are interpreted as within-dog longitudinal descriptors rather than evidence of group-level recovery patterns.

In humans, gait variability is typically increased in musculoskeletal and neurological disorders, is associated with a higher risk of falls in community-dwelling older adults, and reflects disease-specific impairments in neuromuscular control in conditions including osteoarthritis, spinal diseases, and Parkinson’s disease (47–49). Similarly, cyclogram-based studies have revealed that pathological gait is characterized by irregularly dispersed loops that become more organized after intervention (7, 9). These prior observations provide a conceptual rationale for considering stride-to-stride dispersion of cyclogram geometry as a potentially informative descriptor, but its clinical meaning and responsiveness in dogs require validation in larger samples.

From this perspective, the mean value of A_abs reflects the extent to which the available joint configuration space is used by the coordination pattern, whereas the stride-to-stride variability (CV) of A_abs indicates within-session stride-to-stride dispersion (consistency) of the pattern. By capturing both the extent and consistency of inter-joint coordination, A_abs provides a useful metric for monitoring not only the quantitative recovery of movement but also the qualitative stabilization of gait patterns. In parallel, the healthy-cohort repeatability analysis provides SEM and MDC95 estimates (Table 1), which can be used as practical benchmarks when interpreting whether an observed longitudinal change in A_abs is likely to exceed measurement error under standardized acquisition conditions.

### 4.5 Clinical implications and translational potential

In this study, A_abs was calculated based on a small number of strides and required only the sagittal plane joint angle data as input. Because A_abs is computed from the sagittal plane joint angle trajectories, it can, in principle, be derived from kinematic data obtained using either three-dimensional motion capture or two-dimensional video-based analysis. However, cross-modality agreement was not evaluated in this study; therefore, absolute A_abs values should be interpreted within each acquisition method, and longitudinal monitoring should ideally use a consistent acquisition and analysis setup. Consistent with this, our healthy-cohort repeatability analysis indicates that averaging across a standardized set of consecutive cycles can substantially improve measurement stability (Table 1), which is directly relevant to clinical implementation. This indicates that cyclograms and A_abs may be implemented in clinical settings that lack dedicated three-dimensional gait laboratories, provided that basic video capture and simple two-dimensional kinematic analysis are available with standardized video acquisition procedures and appropriate acknowledgment of the limitations of two-dimensional kinematics. Markerless gait analysis technologies are rapidly evolving in human medicine, and similar approaches are beginning to emerge in small animal practice. As such systems become more widely accessible, cyclograms and A_abs may offer a generalizable framework for transforming automatically extracted joint angle trajectories into quantitative and qualitative gait descriptors. In this context, a cancellation-robust total-area definition of A_abs for potentially self-intersecting loops (Section 2.6) may help standardize interpretations of loop “size” across diverse gait patterns.

Structured physiotherapy programs following FHNO have been associated with improved functional outcomes and owner-reported satisfaction, and early initiation of postoperative physiotherapy may further enhance the recovery of limb use (34, 35, 50). Incorporating cyclogram-derived metrics, such as A_abs, into such rehabilitation protocols could provide an objective quantitative framework for tracking inter-joint coordination over time and across institutions, thereby refining clinical decision-making regarding when and how to increase exercise intensity and duration. Nevertheless, in the present manuscript, the FHNO component is intended only as an illustrative feasibility demonstration of within-dog longitudinal monitoring and is not used to infer generalizable postoperative trajectories or treatment effects.

As demonstrated here, anchoring cyclograms to discrete hindlimb gait sub-phases further extends their interpretive value. Rather than merely stating that A_abs has increased or decreased, clinicians may be able to identify which sub-phase(s) of the gait cycle inter-joint coordination is most abnormal based on phase-specific loop segments. In this sense, the proposed framework can be viewed as an attempt to formalize the subjective impressions that experienced clinicians derive from visual gait assessments by expressing them in terms of angle–angle trajectories and phase-annotated loop morphology. Ultimately, combining the mean value and stride-to-stride variability of A_abs with phase-resolved changes in loop morphology could help clinicians determine which joint and part of the gait cycle should be prioritized in rehabilitation strategies and provide an objective basis for evaluating treatment response. Where comparisons across heterogeneous cohorts are unavoidable, ROM-normalized indices (e.g., A_norm) may serve as exploratory tools to reduce scale effects; however, their validity and cross-modality comparability should be established in dedicated studies before clinical thresholds are proposed.

In addition, the qualitative description that “loops are narrow and geometrically compressed immediately after surgery but become larger and more stable as rehabilitation progresses” is intuitively understandable not only by clinicians, but also by dog owners. Presenting cyclograms alongside canonical loops from healthy dogs as a form of visual feedback may enhance owner motivation to continue rehabilitation and strengthen their understanding and acceptance of treatment plans. If the same metrics are adopted across institutions, A_abs and related cyclogram features could also serve as harmonized outcome measures for multicenter case series or intervention trials.

It should be emphasized that the improvements in A_abs and cyclogram morphology observed in this study reflect the combined influence of multiple factors, including the FHNO surgery itself, natural recovery, pain relief, environmental management, and rehabilitation programs. As this was a single-case, uncontrolled, longitudinal observation, the present data did not allow us to disentangle the specific contribution of each component. Accordingly, A_abs and cyclogram analyses should be regarded not as tools for isolating causal effects of individual interventions, but as descriptive indices and a shared visual/quantitative language for monitoring global changes in gait, both quantitatively and qualitatively, at the level of inter-joint coordination. The ability to provide such a common language under relatively simple measurement conditions represents, in our view, a key aspect of the clinical value of this approach. This clinical value is expected to be maximized when acquisition conditions are standardized and when interpretation is anchored to validated repeatability/error estimates (Table 1) and an appropriate reference pattern (Table 2).

### 4.6 Limitations and future directions

This study had several limitations that should be acknowledged. First, only a single case of FHNO was examined in detail. Dogs undergoing FHNO differ widely in body size, breed, underlying pathology, surgical techniques, and postoperative management (including pain control and rehabilitation protocols), and their long-term functional recovery trajectories also vary substantially. Similarly, previous outcome studies have reported a spectrum ranging from dogs with excellent long-term function to those with persistent kinetic asymmetries and residual lameness. Accordingly, the changes in cyclogram morphology and A_abs documented in the present case should be interpreted primarily as an illustrative example of how these metrics can be applied to a postoperative FHNO patient, rather than as findings that can be generalized to all dogs undergoing FHNO. Moreover, because the healthy beagle cohort and the FHNO case differed in breed, body size, walking condition, and data acquisition modality, we deliberately refrained from formal numerical comparisons of A_abs between the cohorts and instead focused on longitudinal changes within the FHNO dog. Specifically, the healthy cohort was evaluated during treadmill walking using a three-dimensional motion capture model, whereas the FHNO case was evaluated during overground walking using a two-dimensional sagittal-plane video approach; these differences can influence estimated joint kinematics and cyclogram geometry and may confound interpretation of between-cohort numerical differences (20). To partially mitigate scale effects, we additionally computed a ROM-normalized index (A_norm); however, given the pronounced between-group heterogeneity, this normalization should be regarded as exploratory and was not used to draw pathology-specific conclusions. Accordingly, any single-case vs. controls discrimination results reported here should be regarded as exploratory and hypothesis-generating, and not as evidence of pathology-specific effects or clinical classification performance. In addition, kinematic analyses were unilateral in both datasets: in the healthy beagle cohort, cyclograms were derived from the left hindlimb only without bilateral recording or side-to-side comparison, and in the FHNO case, only the operated (right) hindlimb was digitized. Unilateral marker placement and analysis have been used in prior canine kinematic studies under controlled experimental conditions (28). Although limb-to-limb differences in hindlimb kinematics in sound dogs may be small under controlled conditions, mechanically meaningful laterality/limb dominance can still be present even in orthopedically normal dogs (51, 52). Therefore, we could not evaluate inter-limb asymmetry or quantify contralateral compensatory inter-joint coordination patterns, which may be clinically relevant during postoperative recovery. Future studies should incorporate bilateral acquisition and symmetry-based analyses to characterize both recovery of the operated limb and compensatory adaptations of the contralateral limb. Future studies, including larger cohorts of diverse breeds, body sizes, and comorbidities, are needed to evaluate the generalizability, robustness, and clinical utility of these measures.

Second, in both healthy beagles and the FHNO case, joint kinematics were obtained using skin-marker-based motion capture methods rather than biplanar fluoroscopy or bone-fixed markers. Skin-marker–based methods in dogs are susceptible to soft tissue artifacts, particularly around the pelvis and hip, and therefore provide less accurate estimates of pelvic and hip kinematics than those of the more distal joints in the sagittal plane (3, 27). Consequently, the absolute values of the joint angles reported here should be interpreted with caution, and the cyclograms and A_abs are best viewed as descriptors of relative inter-joint coordination patterns rather than as precise measurements of true bone motion. In addition, the FHNO case was analyzed using a two-dimensional sagittal-plane approach, which cannot capture out-of-plane (frontal/transverse-plane) motion and may be sensitive to camera alignment and limb rotation; therefore, the derived joint angles and cyclogram geometry should be interpreted as two-dimensional approximations of inherently three-dimensional limb motion. Future studies that rely on biplanar fluoroscopy or advanced markerless three-dimensional tracking will be important to validate whether the cyclogram patterns observed here are robust to soft tissue artifacts. More broadly, cross-modality agreement (3D vs. 2D) and its impact on cyclogram geometry and area indices should be quantified before absolute thresholds are generalized across acquisition systems.

Third, the healthy reference group consisted exclusively of beagle dogs. Beagles are widely used as experimental animals and represent a common model in kinematic research. However, in clinical practice, dogs present with a broad range of limb lengths, body masses, and conformations. These factors are expected to influence the joint angle trajectories and cyclogram geometry. Therefore, the canonical loops presented here should be regarded as breed-specific reference patterns for beagles. In particular, breed- and conformation-related differences have been reported for hip joint kinematics during walking, suggesting that hip-related cyclogram geometry may vary across breeds even under standardized treadmill conditions (40). Future studies should aim to construct normative cyclogram databases across a wide range of breeds, including small-, large-, and short-legged dogs, to enable both between-breed comparisons and the establishment of breed-specific reference values.

Fourth, all gait data analyzed in this study were obtained under a single controlled condition: straight-line walking at a comfortable speed. Both A_abs and the cyclogram shape are likely to be influenced by walking speed and environmental conditions. Thus, the present findings may not be directly applicable to other locomotor tasks such as trotting, running, or walking on inclined or uneven surfaces. A systematic investigation of A_abs and cyclogram features across different speeds and task conditions is required to clarify the speed and condition dependence of these metrics, and to define appropriate standardized testing protocols for clinical and research use.

## 5 Conclusion

This study applied planar hip–stifle and tarsus–stifle cyclograms to the assessment of canine hindlimb gait and demonstrated that cyclograms and A_abs-based indices can be generated not only under laboratory conditions but also in a clinical rehabilitation setting. In healthy beagles, cyclograms yielded reproducible canonical loop patterns that compactly summarized how the hip, stifle, and tarsus coordinated throughout the gait cycle. Reproducibility in healthy beagles was quantified using within-session repeatability statistics [ICC (1,1) and ICC (1,10)] and measurement-error indices (SEM and MDC95) derived from stride-wise A_abs. In the unilateral FHNO case, the enclosed cyclogram area (A_abs) and its stride-to-stride variability captured early postoperative disruption and subsequent partial restoration of inter-joint coordination, supporting the feasibility of within-dog longitudinal monitoring as an illustrative example rather than providing generalizable disease-level inference.

Thus, cyclograms provide an intuitive tool for visualizing how joint motions are combined within a single stride, using the loop shape and area as simple descriptors of coordination. Future studies involving larger numbers of dogs, multiple breeds, and diverse pathologies are warranted to determine the responsiveness, reliability, and prognostic value of cyclogram-derived metrics and to establish their role as objective outcome measures in veterinary physical rehabilitation strategies.

## Data Availability

The raw data supporting the conclusions of this article will be made available by the authors, without undue reservation.
